# Stress Response and Pathogenicity of the Necrotrophic Fungal Pathogen *Alternaria alternata*

**DOI:** 10.6064/2012/635431

**Published:** 2012-12-10

**Authors:** Kuang-Ren Chung

**Affiliations:** ^1^Citrus Research and Education Center, Institute of Food and Agricultural Sciences (IFAS), University of Florida, 700 Experiment Station Road, Lake Alfred, FL 33850, USA; ^2^Department of Plant Pathology, IFAS, University of Florida, Gainesville, FL 32611, USA

## Abstract

The production of host-selective toxins by the necrotrophic fungus *Alternaria alternata* is essential for the pathogenesis. *A. alternata* infection in citrus leaves induces rapid lipid peroxidation, accumulation of hydrogen peroxide (H_2_O_2_), and cell death. The mechanisms by which *A. alternata* avoids killing by reactive oxygen species (ROS) after invasion have begun to be elucidated. The ability to coordinate of signaling pathways is essential for the detoxification of cellular stresses induced by ROS and for pathogenicity in *A. alternata*. A low level of H_2_O_2_, produced by the NADPH oxidase (NOX) complex, modulates ROS resistance and triggers conidiation partially via regulating the redox-responsive regulators (YAP1 and SKN7) and the mitogen-activated protein (MAP) kinase (HOG1) mediated pathways, which subsequently regulate the genes required for the biosynthesis of siderophore, an iron-chelating compound. Siderophore-mediated iron acquisition plays a key role in ROS detoxification because of the requirement of iron for the activities of antioxidants (e.g., catalase and SOD). Fungal strains impaired for the ROS-detoxifying system severely reduce the virulence on susceptible citrus cultivars. This paper summarizes the current state of knowledge of signaling pathways associated with cellular responses to multidrugs, oxidative and osmotic stress, and fungicides, as well as the pathogenicity/virulence in the tangerine pathotype of *A. alternata*.

## 1. Introduction

*Alternaria* species have different lifestyles, ranging from saprophytes to endophytes to pathogens [1]. *Alternaria* species are a highly successful group of fungal pathogens that cause diseases in a wide variety of economically important crops, including apple, broccoli, cauliflower, carrot, citrus, pear, rice, strawberry, tomato, potato, and tobacco, as well as many ornamental and weed species. Due to their wide host range and worldwide distribution, *Alternaria* species cause severe economic problems. *Alternaria *species have been reported to cause diseases in nearly 400 plant species; *A. alternata* alone can infect more than 100 plant species [2–4]. One reason for the success of these pathogens may be attributed to their production of diverse phytotoxins [5, 6]. The host-selective toxins (HSTs) produced by many members of the genus *Alternata* have unique modes of action and toxicity to their respective host plants. Production of HST is critical for successful pathogenesis because HST-deficient mutants are incapable of attacking their host plants [7–11]. In addition to HSTs, many *Alternaria* species produce nonhost selective phytotoxins, such as brefeldin A, altertoxin, and tentoxin [1]. Others can produce mycotoxins that are harmful to humans and other animals [12]. Several *Alternaria *species can also cause upper respiratory tract infections and asthma in humans [13].

**Figure 1:**
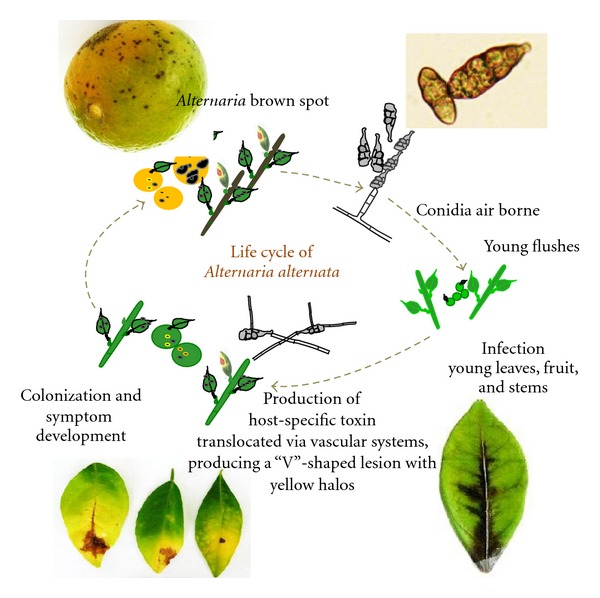
Life cycle of *Alternaria alternata*, the causal agent of citrus brown spot. ACT toxin produced by the tangerine pathotype of *A*. *alternata* is transported via the vascular system and formation of necrotic lesions on a detached calamondin leaf (bottom right).

*Alternaria alternata* (Fr.) Keissler has several pathogenic variants, each producing a unique HST and causing disease in different host plants [5, 9, 10, 14, 15]. HSTs produced by *Alternaria *pathotypes are chemically diverse, ranging from low-molecular-weight compounds to cyclic peptides. The genes encoding polypeptides for biosynthesis of *Alternaria* HSTs have been shown to reside on a dispensable chromosome [9]. In citrus, *A. alternata* has two major pathotypes—the tangerine pathotype and the rough lemon type [16]. The citrus pathotypes are morphologically similar and can be differentiated only by pathological and genetic analyses [17]. The rough lemon pathotype, producing the host-selective ACRL toxin, is pathogenic exclusively to lemon (C*itrus jambhiri* Lush) and Rangpur lime (*Citrus x limonia* Osbeck). ACRL toxin affects mitochondrial function, disrupting posttranscriptional RNA splicing and causing metabolite leakage and malfunction of oxidative phosphorylation in susceptible host cells [18, 19]. In contrast, the tangerine pathotype of *A. alternata* produces the host-selective ACT toxin with a core 9,10-epoxy-8-hydroxy-9-methyl-decatrienoic acid structure [20] and causes brown spots on citrus leaves and fruit. ACT toxin is highly toxic to tangerines (*C. reticulata* Blanco) and grapefruit (*C. paradisi* Macfad.), as well as hybrids from grapefruit and tangerine, or tangerine and sweet orange (*C. sinensis* Osbeck). ACT toxin does not affect rough lemon or Rangpur lime [20]. The toxin is quickly translocated outward through the vascular system, causing rapid electrolyte leakage and necrotic lesions along the veins (Figure 1). *A. alternata* infection in citrus leaves induces rapid lipid peroxidation and accumulation of hydrogen peroxide (H_2_O_2_) [21]. Studies show that *A. alternata* has evolved a dramatic flexibility and uniqueness in the signaling pathways in order to respond to diverse environmental stimuli and to thrive within host plants. This paper discusses signaling pathways related to oxidative and osmotic stress resistance, fungicide sensitivity, conidia formation, and pathogenesis of *A. alternata*.

## 2. Roles of Reactive Oxygen Species in Plant-Fungal Interactions

All organisms with an aerobic lifestyle inevitably generate toxic reactive oxygen species (ROS), primarily superoxide (O_2_^−^), and hydrogen peroxide (H_2_O_2_) during physiological metabolisms [22–26]. During the course of host colonization, fungal pathogens of plants need to overcome a wide range of potentially harmful environmental challenges, particularly an oxidative burst, which could result in the production and accumulation of highly toxic ROS. In addition to the direct toxicity of ROS to cells, when produced in abundance, ROS can also serve as secondary messengers in the pathogen-response signal transduction pathways [23, 27]. Among ROS, H_2_O_2_ is relatively stable and able to pass freely through membranes, serving as a signaling cue for defense responses in surrounding cells and as a substrate for oxidative cross-linking in the plant cell wall [27–32]. Hydrogen peroxide can react with O_2_^−^ via the Haber-Weiss reaction or with metal ions via the Fenton pathway [33–35] to generate the extremely toxic hydroxyl radical. It has been well known that plants produce toxic ROS as a defense against pathogens [36–41]. In response to the microbe invasion, plant cells often produce excessive amounts of H_2_O_2_ by a specific plasma membrane NADPH oxidase, termed as the hypersensitive reaction (HR), which leads to programmed cell death and cellular defense against pathogen attack [42–46]. The HR plays a vital role in plant defenses against saprophytes and noncompatible or biotrophic pathogens; however, HR has been shown less effective against necrotrophic phytopathogens [47–51]. ROS have been shown to be involved in nonhost resistance in plants as well [52, 53]. 

The burst of the HR may ironically provide an advantage for necrotrophic phytopathogens, as they acquire nutrients exclusively from dead cells [54]. ROS have been thought to enhance plant colonization by necrotrophic pathogens such as *Botrytis cinerea* and *Sclerotinia sclerotiorum* [50, 51, 55]. Because many necrotrophic pathogens are able to produce a wide array of HSTs or cell-wall-degrading enzymes that kill host cells before colonization, leading to the accumulation of ROS, the pathogens must have evolved effective mechanisms to cope with the toxicity of ROS [47, 49].

## 3. YAP1-Mediated ROS Detoxification in *A. alternata*

ROS damage a wide range of biological molecules, including fatty acids, proteins/enzymes, sugars, and nucleic acids; thus, exposure to ROS may result in cell death [56–58]. The relative sensitivity of the fungal pathogen to ROS is likely determined by the effectiveness of its own ROS detoxification ability. In order to survive under aerobic conditions, fungi must have detoxification systems that can effectively scavenge ROS, maintain reduced redox states within subcellular microenvironments, and repair ROS-triggered damage [22, 59, 60]. Molecular and genetic studies aimed at understanding the mechanisms by which cells cope with the oxidative stresses and are protected from the deleterious effects of ROS have been intensively studied in both prokaryotes and eukaryotes. In the budding yeast *Saccharomyces cerevisiae*, the YAP1 transcription regulator plays a central role in the cellular pathways associated with the oxidative stress response [61, 62]. YAP1 is responsible for transcriptional activation of genes involved in multidrug resistance as well. YAP1, resembling mammalian AP-1, has a basic leucine zipper (bZIP) domain and has been shown to be activated by H_2_O_2_ and various ROS-generating oxidants, as well as heavy metals [63–66]. In the absence of oxidative challenges, YAP1 can be found in the cytoplasm at low levels. Upon perceiving oxidative or chemical stimuli, YAP1 quickly forms disulfide bonds, changes conformation, and is translocated into the nucleus where YAP1 regulates the expression of genes responsible for stress alleviation [67–71]. Conserved cysteine residues in both the amino and carboxyl terminal domains are essential for the formation of the disulfide bonds, nuclear relocalization and transcriptional regulation of YAP1 [72]. 

Although all microorganisms employ complex mechanisms, both enzymatic and nonenzymatic to avoid ROS toxicity [47, 61, 73], the pathological roles of oxidative stress mitigation remain uncertain in pathogenic fungal species. The role of ROS in host resistance and pathogen invasion is likely dictated by the physiological conditions of the host, the lifestyle of the pathogen, and the combination of different stimuli [38, 41, 74–76]. Hence, ROS produced by plants may have different effects against different pathogens [49, 75]. YAP1-mediated detoxification of ROS is an essential virulence determinant in the opportunistic human pathogen *Candida albicans* and the biotrophic maize pathogen *Ustilago maydis* [77, 78]. However, YAP1 is not required for virulence in the plant pathogens *Cochliobolus heterostrophus* and *B. cinerea* and in the animal pathogen *Aspergillus fumigatus*, even though the disrupted mutants exhibit increased sensitivity to H_2_O_2_ [79, 80]. The necrotrophic fungus *Sc. sclerotiorum* produces oxalic acid that suppresses host-generated ROS, and thus facilitates fungal evasion [73]. The *Magnaporthe oryzae MoHYR1* gene encoding a glutathione peroxidase (GSHPx) is required for detoxifying plant-generated ROS and full virulence [81]. In *S. cerevisiae*, HYR1 forms a disulfide bond with YAP1, inducing a conformational alteration and nuclear localization of YAP1 upon exposure to ROS [82].

**Figure 2:**
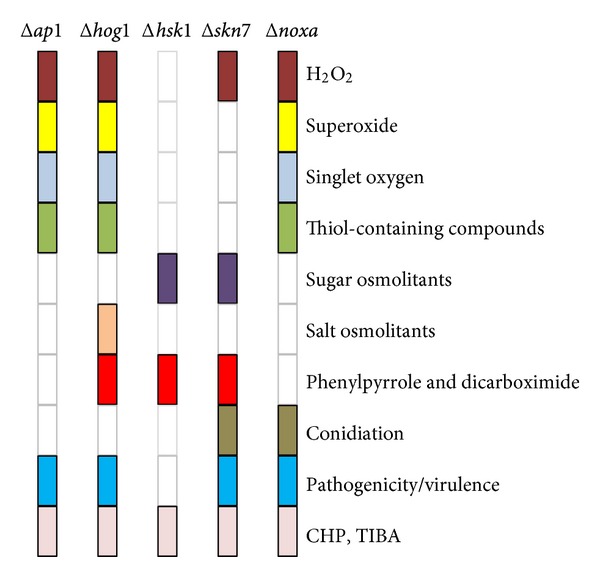
Phenotypic changes in *A*. *alternata* mutants lacking the transcription regulator (*YAP1*), the MAP kinase (*HOG1*), the “two-component” histidine kinase (*HSK1*), the response regulator (*SKN7*), or the NADPH oxidase (*NOXA*). Open rectangles denote wild-type phenotypes.

To thrive within host plants, *A. alternata* must be able to detoxify or obviate the ROS-mediated plant defense barriers. Our studies have demonstrated that cellular detoxification of ROS regulated by the redox-responsive YAP1 transcription regulator is important for pathogenesis of *A. alternata* to citrus [83, 84]. Inactivation of the *A. alternata AP1* gene (designated *AaAP1*), encoding a YAP1-like transcription factor, resulted in fungal mutants that are hypersensitive to H_2_O_2_, menadione, and potassium superoxide (KO_2_). The promoter of *AaAP1* contains a putative stress responsive element (STRE: AGAGGGG). Upon activation by H_2_O_2_, the AaAP1::sGFP fusion protein became localized in the nucleus. Fungal mutants lacking *AaAP1 *(Δ*yap*1) are weakly virulent on susceptible citrus cultivars even though they synthesize HST toxins normally. However, Δ*yap*1 mutant is not sensitive to osmotic and salt stress-related compounds (e.g., sorbitol, mannitol, NaCl, and KCl) (Figure 2). Δ*yap*1 mutant produces wild-type level of conidia that germinate at a rate and magnitude similar to the wild-type strain. The nonpathogenic phenotype of Δ*yap*1 mutant was accompanied with reduced activities of fungal antioxidants, including catalase, peroxidase, superoxide dismutase (SOD), and glutathione reductase. The inability of Δ*yap*1 mutants to incite necrotic lesions is likely due to the mutants' inability to detoxify ROS because coapplication of Δ*yap*1 mutants with the NADPH oxidase inhibitor, apocynin, or diphenylene iodonium, partially restored lesion-forming capability to the mutants. Δ*yap*1 mutant is impaired in the penetration and colonization stages because the impaired mutant did not cause any visible necrotic lesions on wounded or unwounded leaves of the citrus cultivar Minneola. All mutant phenotypes were completely restored to the wild type in fungal strains expressing a functional copy of *AaAP*1. Δ*yap*1 mutant displayed severe defects in antioxidant activities and was unable to detoxify H_2_O_2_ effectively. Our studies concluded that effective detoxification of ROS via the AaAP1-mediated pathway is absolutely required for successful colonization of citrus by *A. alternata* [83, 84]. The tobacco pathotype of *A. alternata* impaired for the biosynthesis of mannitol, an antioxidant and quencher of the hydroxyl radical, also greatly reduces virulence [85, 86], consistent with the importance of ROS detoxification in the pathogenesis of *A. alternata*. 

Furthermore, *AaAP*1 was found to be required for full resistance to 2,3,5-triiodobenzoic acid (TIBA), 2-chloro-5-hydroxypyridine (CHP), diethyl maleate (DEM), and many pyridine-containing compounds [87]. Diethyl maleate is a glutathione-depleting agent that has been shown to generate a nonreversible modification of cysteine residues in the *Schizosaccharomyces pombe* Pap1 protein. As a result, Pap1 is constitutively localized in nucleus and activates the genes required for ROS tolerance [88]. TIBA is often used as herbicides or as an inhibitor of indole-3-acetic acid (IAA) transportation [89, 90]. Pyridine is a heteroaromatic compound composed of five carbons and one nitrogen atom. Pyridine could accelerate the production of superoxide and hydroxyl radicals when Cu^2+^ and H_2_O_2_ are present [91, 92]. Pyridine and its derivatives serve as constituents of RNA and DNA, as electron carriers such as NADP/NADPH and flavin nucleotides (FAD/FADH) and as energy storage molecules such as ATP and GTP. 

## 4. The “Two-Component” Histidine Kinase (HSK) Signaling Pathway 

All living cells have a complicated yet well-regulated network often comprising different signaling transduction pathways to perceive changes in their environments and to adjust physiological and developmental processes [93–99]. “Two-component” histidine kinase (HSK) signaling transfer systems are commonly present in bacteria, slime molds, fungi, and plants; however, these systems have not yet been identified in animals [100, 101]. In bacteria, HSK signaling systems contain a histidine kinase (HSK) and a response regulator (RR); each is encoded by a separate gene [102, 103]. In contrast, all fungal HSKs have both the HSK and RR domains [101, 104, 105]. In response to environmental changes, a series of phosphate transfers between histidine (His) and aspartate (Asp) residues occurs in a pattern of His-Asp-His-Asp to regulate downstream signaling pathways such as mitogen-activated protein kinase (MAPK) cascades and eventually leads to a change in gene expression [100, 106, 107]. 

**Figure 3:**
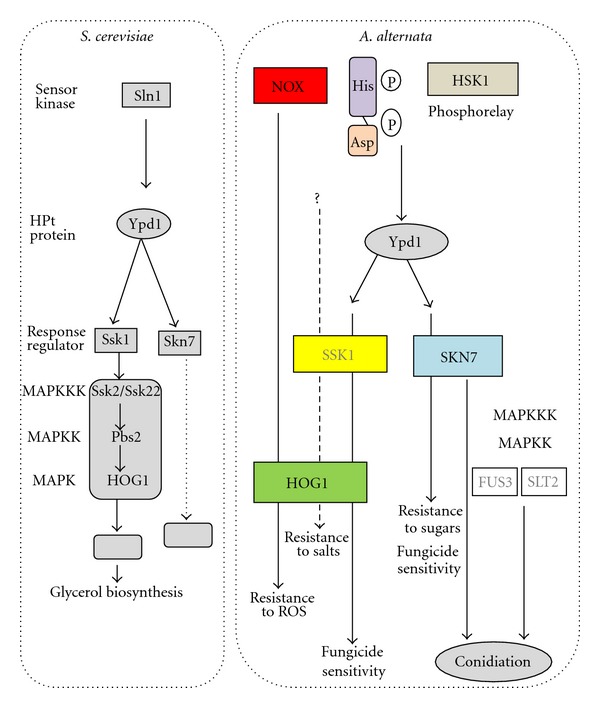
Schematic illustration and comparison of signaling pathways leading to osmotic stress resistance in the budding yeast, *S*. *cerevisiae*, and the pathways leading to ROS resistance, osmotic stress response, fungicide sensitivity, and conidia formation in the tangerine pathotype of *A*. *alternata*.

The *S. cerevisiae* histidine kinase, designated SLN1p, is required for osmotic adaption via the SLN1p-YPD1p (a protein containing a His phosphotransfer domain)-SSK1p or SKN7p cascade [108, 109]. SSK1p is the major regulator for osmolarity response; SKN7p plays only a minor role in osmosensing. Under normal osmolarity, the SLN1p kinase is phosphorylated and able to activate YPD1p and SSK1p with a phosphorelay mechanism (Figure 3). The phosphorylated SSK1p is inactive and incapable of activating the High Osmolarity-Glycerol 1 (HOG1) MAP kinase pathway (see below for details). In contrast, SLN1p is not phosphorylated under conditions of high osmolarity; therefore SSK1p is able to activate the HOG1-signaling cascade. The activated HOG1 pathway is responsible for glycerol accumulation, allowing the yeast to cope with the high osmolarity. *S. cerevisiae* also utilizes a non-HSK-related protein SHO1p to cope with osmotic stress [108, 110]. However, deletion of an *SHO1* homolog in *A. alternata* did not impact cellular tolerance to oxidative and osmotic stress, fungicide sensitivity or fungal virulence (L.-H. Chen, unpublished). 

The budding yeast *S*. *cerevisiae* has only one HSK; all other fungi have multiple HSK signaling genes [111]. Fungal HSKs are divided into 11 groups based on phylogenetic relationships inferred from the conserved HSK and RR domains. Among them, Group III HSK is one of the best characterized HSKs in the filamentous fungi. Collectively, Group III HSK has been implicated in osmotic and oxidative responses, toxin biosynthesis, hyphal development, conidia formation, and virulence, as well as sensitivity to dicarboximide and phenylpyrrole fungicides in different fungal species [112–121]. 

Signals sensed by HSK are often transduced down to the HOG1 MAP kinase pathway. Fungi lacking Group III HSK or HOG1 often became resistant to dicarboximide and phenylpyrrole fungicides and exhibited an elevated sensitivity to osmotic stress [117, 122, 123]. Although the HSK-HOG1 signaling pathway is conserved, it may be recruited for divergent functions in different fungal species. As discussed above, the *S. cerevisiae* SLN1p negatively regulates HOG1 phosphorylation under conditions of high osmolarity [100, 106, 107]. The filamentous fungus *Co. heterostrophus* Group III HSK (Dic-1) positively regulates phosphorylation of the HOG1 MAP kinase, which subsequently activates expression of genes responsible for osmotic resistance and fungicide sensitivity [117]. In *B. cinerea*, the HOG1-like MAP kinase is not required for fungicide sensitivity even though it is negatively regulated by the “two-component” HSK. Furthermore, the salt-tolerant yeast species, *Hortaea werneckii*, copes with osmotic stress using a Group VII HSK-HOG1 pathway [124]. Those studies indicate that the HSK-HOG1 signaling pathways can be operated in very different regulatory mechanisms in various species.

The *AaHSK1 *gene, encoding a putative histidine kinase, was cloned from the tangerine pathotype of *A. alternata *[87]. AaHSK1, containing no transmembrane regions, is required for adaption to osmotic stress induced by sugars but not by salts (Figure 2). Δ*hsk*1 displayed increased sensitivity to glucose, sucrose, sorbitol, or mannitol, but not to H_2_O_2_, KCl, or NaCl [125]. Similarly, the *M. grisea *HSK is required for resistance to sugar, but not salt, osmotic stress [116]. The *F. oxysporum* histidine kinase Fhk1 is responsible for resistance to osmotic stress, menadione, but not H_2_O_2_ [126]. Similar to Δ*yap*1 mutant, *AaHSK1 *disruption mutants displayed an elevated sensitivity to TIBA and CHP, suggesting a possible link between YAP1 and HSK. Δ*hsk*1 mutants displayed an elevated resistance to dicarboximide (iprodione and vinclozolin) and phenylpyrrole (fludioxonil) fungicides, suggesting that AaHSK1 is one of the primary targets of these fungicides. Similarly, resistance to dicarboximide and phenylpyrrole fungicides has been demonstrated to be associated with a mutation within the gene encoding a Group III HSK and/or an HOG1 MAP kinase in a number of filamentous fungi [113–118, 121, 122, 126, 127]. However, HSK is not involved in dicarboximide susceptibility in *A. longipes*.

The *A. alternata *HSK1 is not required for response to oxidative stress. The *AaHSK1 *gene product is not involved in pathogenicity or virulence because the *AaHSK1*-impaired mutants (Δ*hsk*1) induced necrotic lesions at rates and magnitudes similar to the wild-type strain or the genetically reverted strain on wounded or unwounded leaves of citrus. In contrast, Group III HSK is a virulence determinant in the phytopathogenic fungi *B. cinerea*, *Claviceps purpurea*, and *Fusarium oxysporum* and in the human pathogen *Cryptococcus neoformans* [118, 126, 128, 129]. 

## 5. The HOG1 Mitogen-Activated Protein kinase- (MAPK-) Mediated Signaling Pathway 

The HOG1 MAPK-mediated signaling cascades in eukaryotic cells are vital for sensing environmental stimuli and for transmitting these signals to the nucleus to modulate gene expression [130, 131]. MAPK-mediated cascade pathways are composed of three serine/threonine protein kinases—MAP kinase kinase kinase (MAPKKK), MAP kinase kinase (MAPKK), and MAP kinase (MAPK). This signal transduction pathway, in conjunction with HSK, is well conserved in all eukaryotes and functions in perceiving environmental stimuli via phosphorylation and gene activation [132, 133]. The phosphorylated MAPK activates a set of genes via regulating appropriate transcription factors. 

The *A. alternata* ortholog (AaHOG1) contains a distinct phosphorylation motif (TGY) involved in the osmotic stress response [134]. Inactivation of the HOG1 ortholog by targeted gene disruption in the tangerine pathotype of *A. alternata* resulted in mutants that are highly sensitive to the oxidants *tert*-butyl-hydroxyperoxide, H_2_O_2_, and menadione, salts (Figure 2), as well as TIBA and CHP [87]. Because fungal strain lacking the FUS3 MAP kinase (Δ*fus*3) grew faster than wild type in the presence of KCl or NaCl [125]. Thus, AaHOG1 and FUS3 play an opposite role in KCl and NaCl tolerance. HOG1 has been shown to suppress the FUS3/KSS1 signaling cascade during hyperosmotic stress in *S. cerevisiae* [135, 136]. *A. alternata* strains impaired at *AaHOG1 *(Δ*hog*1) displayed wild-type levels of sensitivity to high concentrations of glucose, sucrose, sorbitol, or mannitol (Figure 2) even though sugar osmoticants increased AaHOG1 phosphorylation and subsequently nuclear localization in the Δ*hsk*1 mutant background. In the wild-type background, sugar osmoticant had less effect on AaHOG1 phosphorylation and did not facilitate nuclear localization of AaHOG1. 

The wild-type isolate of *A. alternata* is extremely sensitive to dicarboximide and phenylpyrrole fungicides, whereas fungal strain lacking *AaHSK1 *is highly resistant to them. Compared to the resistance seen in mutants defective at *AaHSK1*, Δ*hog*1 mutant displayed only slightly increased resistance to these fungicides. In the wild-type strain of *A. alternata*, the AaHOG1 protein was phosphorylated at low levels under normal conditions. Exposure to iprodione or fludioxonil fungicide, NaCl, or H_2_O_2_ elevated AaHOG1 phosphorylation to varying degrees. Although impairment of *AaHSK1* reduces AaHOG1 phosphorylation, *A. alternata* apparently recruits AaHSK1 and AaHOG1 to exert a unique function in resistance to sugar osmoticants and salt stress, respectively (Figure 3). 

Under unchallenged conditions, expression of AaHOG1::sGFP fusion protein under control of the endogenous *AaHOG1 *promoter in the wild-type strain resulted in green fluorescence uniformly diffused along the hyphal cytoplasm. However, the green fluorescence became dense patches after exposure to H_2_O_2_, iprodione and fludioxonil fungicides, or NaCl. Thus, nuclear localization is important for proper functions of HOG1. Compared to Δ*hsk*1 or Δ*yap*1 mutant, Δ*hog*1 mutant was highly resistant to cell wall-degrading enzymes (lyticase, driselase, *β*-D-glucanase, and *β*-glucuronidase), thereby failing to generate any protoplasts. As judged from distinct phenotypes in fungal mutants impaired in the *AaHSK1* or *AaHOG1* gene in *A. alternata*, it appears that AaHSK1 functions in osmotic tolerance and fungicide sensitivity via AaHOG1 and other gene (e.g., SKN7) activation branches. 

AaHSK1 plays no role in pathogenesis of *A. alternata*. On the other hand, pathogenicity assays revealed that the *AaHOG1*-impaired mutants are nonpathogenic, producing no necrotic lesions on Minneola leaves that were unwounded or prewounded before inoculation. Similar to *AaAP1 *disruption, the *AaHOG1*-impaired mutant is defective at the penetration and colonization steps. Inactivation of the *AaHOG1 *gene did not impact the production of host-selective ACT toxin by *A. alternata*. HOG1 is required for virulence/pathogenicity in various fungal pathogens. These include *Co. heterostrophus*, *Cryphonectria parasitica*, *B. cinerea*, *Mycosphaerella graminicola*, *Ca. albicans*, and *C. neoformans* [137–142]. However, HOG1 is not a virulence determinant in *M. grisea*, *Colletotrichum lagenarium*, *Bipolaris oryzae* and *As. fumigatus* [122, 143–145]. Again, a conserved protein may have very different functions in fungi. 

## 6. SKN7-Mediated ROS Detoxification 

 “Two-component” HSK-mediated signal transduction is vital for sensing and adapting to environmental changes in microorganisms. In *S. cerevisiae*, SLN1 histidine kinase transmits signals via a phosphotransfer process down to two response regulators, SSK1p and SKN7p, in response to osmotic stress. However, SKN7p is not regulated by the SLN1p-mediatd phosphorylation in the oxidative stress response [146], indicating that there are two different activation mechanisms in response to osmotic and oxidative stress. Under oxidative stress, SKN7p is phosphorylated at serine or threonine residue, forming a heterodimer with YAP1; together they transcriptionally activate the genes involved in the oxidative stress response [146–152]. YAP1 regulates cadmium resistance independent of SKN7p [153]. SKN7p can also form a heterodimer with the heat-shock transcription factor, the cell cycle transcription regulator, the calcium responsive activator, or the Rho1 GTPase [154–157]. 

In filamentous fungi, SKN7 is required for oxidative stress adaptation, hypoosmotic stress response, cell cycle, sexual mating, sporulation, cell wall biosynthesis, and fungicide sensitivity [120, 146, 155, 158–163]. The *A. alternata SKN7 *homolog (*AaSKN7*) was cloned and characterized in the tangerine pathotype [164]. The promoter of *AaSKN7* contains a putative *st*ress *r*esponsive *e*lement (STRE: AGAGGGG) that is often present in genes induced by various stresses such as oxidative damage in yeasts. AaSKN7 has a heat-shock transcription factor- (HSF-) type helix-turn-helix DNA-binding domain signature and a response regulatory (RR) domain. Genetic mutation analysis revealed that *AaSKN7 *is required for resistance to osmotic and oxidative stress and fungicide sensitivity, as well as conidiation and conidia morphology. AaSKN7 is primarily localized in the nucleus, whereas YAP1 and HOG1 are quickly transported into the nucleus upon sensing oxidative stress. AaSKN7 may interact directly with AaAP1 in nucleus in response to oxidative stress as demonstrated in the budding yeast [151]. Both *A. alternata* AP1 and HOG1 are required for resistance to different types of ROS including hydrogen peroxide, superoxide, and singlet oxygen. On the other hand, AaSKN7 is required for resistance to H_2_O_2_, *tert*-butyl hydroperoxide, and cumyl peroxide, but not to superoxide-generating compounds— diamide, menadione and potassium superoxide (Figure 2). It appears that AaSKN7 and AaHOG1 contribute independently to oxidative stress in *A. alternata*. Because AaHSK1 is not required for ROS resistance [87]; activation of AaSKN7 and AaHOG1 in response to ROS is likely mediated by unknown regulatory sensors other than AaHSK1. Furthermore, *A. alternata* HSK1 apparently can recruit SKN7 and HOG1 to deal with sugar and salt osmoticants, respectively. Although AaHSK1 and AaSKN7 play no roles in resistance to salt-induced stress, AaSKN7 is involved in resistance to sugar osmoticants likely via the AaHSK1—mediated signaling pathway (Figure 3). Δ*skn*7/Δ*hog*1 double mutants were hypersensitive to both salts and sugars, indicating that *A. alternata* is capable of sensing different environmental stimuli using distinct or shared signaling pathways. Furthermore, AaSKN7, independent of AaHSK1, is involved in conidia formation. Our studies also revealed that formation of conidia by *A. alternata *is closely regulated by the FUS3 and SLT2 MAP kinases-mediated signaling pathways, as well as by the G-protein and the NOX complex [125, 165–167]. However, recent studies revealed that cAMP-dependent protein kinase A (PKA) suppresses conidia formation by the tangerine pathotype of *Alternaria alternata* [168]. It remains uncertain if these signaling pathways actually interact during conidia formation.

*A. alternata* strains deleted for *HSK1 *or *HOG1* showed an elevated resistance to dicarboximide and phenylpyrrole fungicides. Δ*skn*7 mutant displayed an elevated resistance to those fungicides at levels between Δ*hsk*1 and Δ*hog*1 mutant strains, indicating that the involvement of SKN7 in fungicide sensitivity is likely mediated by the HSK1 signaling pathway. Fungal strain carrying *skn7/hog1* double mutations exhibited fungicide resistance, similar to the strain carrying a single *AaHSK1 *gene mutation. The results indicated that the signals associated with fungicide sensitivity are transduced from AaHSK1 simultaneously down to both AaSKN7- and AaHOG-mediated pathways. The HSK-HOG signaling pathways are associated with fungicide susceptibility in *A. brassicicola* and *N. crassa* [121]; yet SKN7 is not involved in fungicide sensitivity in *N. crassa*. HSK governs Ssk1p (an upstream regulator of HOG1) and Skn7p for osmolarity adaption and fungicide sensitivity in the phytopathogenic fungus *Co. heterostrophus* and the human pathogen *C. neoformans* [128, 169, 170]. 

The roles of SKN7 in pathogenicity/virulence vary among fungal pathogens. *A. alternata* SKN7 is required for fungal colonization and lesion development in susceptible cultivars of citrus. Similar to Δ*yap*1 and Δ*hog*1, mutational inactivation of *AaSKN7 *in *A. alternata* resulted in reduced activities of catalase, SOD, and peroxidase, confirming further that the ability to detoxify host-generating H_2_O_2_ by *A. alternata *is crucial for successful pathogenesis in citrus. The *SKN7* homologs are required for virulence in the human pathogens *C. neoformans*, *Ca. albicans,* and *Ca. glabrata* [150, 171, 172]. Again, in contrast, *SKN7* is not a virulence determinant in the plant pathogens *Co. heterostrophus* and *M. oryzae* and in the human pathogen *As. fumigatus* [116, 162, 173]. 

## 7. The NADPH Oxidase (NOX): Mediated Signaling Pathway 

The NADPH-dependent oxidase transfers electrons from NADPH to the oxygen molecule, leading to the production of a superoxide that is further metabolized to H_2_O_2_ by SOD [174, 175]. NOX complex is commonly found in animals, plants, and many multicellular microorganisms, but completely absent in prokaryotes [176]. Functionally, NOX complex plays a crucial role in cellular differentiation and defence response. In humans, the phagocytic NOX complex, involved in the production of superoxide and immunity, contains two major catalytic components gp22^phox^ and gp91^phox^ and multiple regulatory subunits Rac (a small GTPase), p40^phox^, p47^phox^, and p67^phox^ [177]. NOX complex is also required for the regulation of hormone responses, cell proliferation, and apoptosis in animals ([178–180]. Activation of gp91^phox^ is primarily regulated by p67^phox^ and Rac2 [181]. Plants also have oxidases analogous to gp91^phox^, designated respiratory burst homologs (Rboh), which are required for physiological metabolisms and for ROS generation in response to pathogen invasion [175, 182, 183]. 

Many fungi have NADPH oxidase orthologs, NOXA, NOXB and NOXC that have been documented by genetic analysis to be required for developmental, physiological and pathological functions [25, 76, 184, 185]. Both NOXA, and NOXB are analogous with mammalian gp91^phox^. Expression of the NOXA/NOXB coding genes is closely regulated by the regulatory subunit, NOXR (p67^phox^ homolog), and the small GTPase (Rac homolog) [185]. Fungal NOXC contains a calcium-binding EF-hand motif and is analogous to the mammalian NOX5 and the plant Rboh enzymes.

The functions of NOX complex in the regulation of multicellular development and pathogenicity vary markedly among fungal species that possess it [186]. Both NOXA and NOXB are involved in the regulation of sclerotia formation in both *B. cinerea *and *Sc. sclerotiorum* [141, 187]. In *B. cinerea*, only NOXB is required for the formation of the penetration structure, even though both NOX isoforms have a role in pathogenicity. In *M. grisea*, NOXA, and NOXB play a role in pathogenesis because both isoforms are required for the formation of penetration peg under the appressorium [188]. While NOXA is required for the development of sexual fruiting body in fungi, only NOXB is required for ascospore germination in *N. crassa* and *Podospora anserina *[189–191]. NOXA, but not NOXB, is required for establishing the mutualistic association between the fungal endophyte *Epichloë festucae* and perennial ryegrass [76]. When inoculated into its grass host,* E. festucae* strain lacking NOXA or NOXR becomes pathogenic, showing increased branching and causing severe stunting and premature senescence of the host [192, 193]. NOXA is coordinately regulated by the small GTPase Rac and NOXR as evidenced by yeast two-hybrid and pull-down analyses [194]. Furthermore, the yeast polarity protein orthologs, Bem1 and Cdc24, have recently been proven to be parts of fungal NADPH oxidase complex [195]. 

The tangerine pathotype of *A. alternata* has *NOXA*, *NOXB*, and *NOXR* homologs. *A. alternata NOXA* (*AaNOXA*) contains a NADPH-binding domain and six transmembrane domains and a ferredoxin synthase-type FAD-binding domain, commonly found in the NOXA-like family. Genetic analysis revealed that *AaNOXA* is responsible for producing superoxide and H_2_O_2_. Δ*noxA* mutants accumulated less ROS within hyphae than the wild type, as judged by nitroblue tetrazolium (NBT), 3,3′-diaminobenzidine (DAB), and dichlorodihydrofluorescein diacetate (H_2_DCFDA) staining for the presence of superoxide and H_2_O_2_. Moreover, deletion of *AaNOXA* in *A. alternata* resulted in an elevated sensitivity to H_2_O_2_, superoxide-generating compounds (menadione and KO_2_), diamide, SDS, CHP, TIBA, and potent singlet oxygen-generating compounds (hematoporphyrin and rose Bengal) (Figure 2). These deficiencies are similar to the phenotypes previously seen for Δ*yap*1 or Δ*hog*1 mutant. Expression of the *AaAP1* and *AaHOG1* genes is likely regulated by AaNOXA, as deletion of *AaNOXA* decreased the accumulation of the *AaAP1* and *AaHOG1* gene transcripts. Reintroducing and expressing a wild-type *AaNOXA *in a Δ*noxA* mutant restored ROS resistance and expression of both *AaAP1* and *AaHOG1* genes. Δ*noxA* mutants also displayed increased sensitivity to NADPH oxidase inhibitors [diphenylene iodonium (DPI) and apocynin], NO^•^-generating compounds [sodium nitroprusside (SNP) and hydroxyl amine HCl (HAD)], NO^•^ synthase substrate (L-arginine) and NO^•^ synthase inhibitor [nitroarginine methyl ester (nitro-arg)]. Similar to Δ*yap*1 and Δ*hog*1, Δ*noxA* mutants, producing normal ACT toxin, induced significantly smaller and fewer necrotic lesions than the wild type on detached Minneola or calamondin leaves 3 days postinoculation, indicating that *NOXA* is an important virulence determinant in *A. alternata*. 

NOXA, NOXB, and NOXR are core components of the NOX complex, responsible for the production of H_2_O_2_. All three NOX components are required for vegetative growth, conidiation, resistance to oxidative and nitrosative stress, and full virulence. However, each isoform may independently and cooperatively interact with other yet unidentified components under different environmental conditions and during different developmental stages because the degree of impairment varied considerably among individual Δ*nox* mutants. Δ*noxA* mutant was more sensitive to H_2_O_2_, KO_2_, and diamide than Δ*noxB* or Δ*noxR*. In contrast, Δ*noxB* or Δ*noxR* mutation strain was more sensitive to cumyl H_2_O_2_ and SDS than Δ*noxA*. The elevated sensitivity of Δ*noxB* and Δ*noxR* mutants to ROS was also accompanied by a reduced expression of two redox-responsive genes *AaAP1* and *AaHOG1*. Although expression of *AaAP1* and *HOG1* was upregulated by the NOX system; both AaAP1 and HOG1 negatively regulate the expression of *NoxB* and *NoxR*. This transcriptional feedback loop might allow fungus to avoid excessive production of toxic ROS. In *A. nidulans*, *NOXA* is regulated by an *HOG1* homolog [189]. Expression of the NOX complex coding genes has been shown to be regulated by FUS3/KSS1 and SLT2 MAP kinases in *N*. *crassa* and *B. cinerea* [141, 191]. Mammalian p47^phox^ and p67^phox^ are phosphorylated by the p38 HOG1 MAP kinase.

In fungi, expression of *NOXA* and *NOXB* is regulated by NOXR and Rac [141, 191, 194]. However, *A. alternata* NOXR negatively regulated the expression of *NOXA *and had no effects for the expression of *NOXB*. Δ*noxB* mutant was highly resistant to calcofluor white, Congo red and dicarboximide and phenylpyrrole fungicides compared to the wild type or the mutant strain lacking *NOXA* and *NOXR*. NOXB seemingly plays a negatively regulatory role in the biosynthesis of chitin because Δ*noxB*, but not Δ*noxA* and Δ*noxR*, had higher chitin content than the wild type (S.L. Yang, personal communication). As stated above, fungal strains disrupted at any of the *AaAP1*, *AaHSK1*, and *AaHOG1* genes were all hypersensitive to CHP and TIBA. Δ*noxA*, Δ*noxB*, and Δ*noxR* mutants also displayed increased sensitivity to these two compounds, suggesting the existence of essential cross-talks between different signaling pathways in the context of multidrug resistance. 

The NOX complex has been shown to be required for pathogenicity/virulence in a number of fungal species [141, 167, 188, 196]. Pathogenicity assays revealed that Δ*noxB* or Δ*noxR* mutant is unable to produce necrotic lesions on unwounded citrus leaves. Both mutants induced wild-type lesions on citrus leaves that were wounded before inoculation, indicating that Δ*nox* mutants are primarily arrested in the penetration stage. Δ*yap*1 and Δ*hog*1 mutants are blocked in both penetration and colonization stages. 

## 8. Nonribosomal Peptide Synthetase- (NPS-) Mediated ROS Detoxification

Deletion of an *A. alternata *gene (*AaNPS6*), encoding a polypeptide analogous to fungal nonribosomal peptide synthetases (NPSs) resulted in fungi that reduced accumulation of host-selective toxin and melanin and displayed increased sensitivity to H_2_O_2_, superoxide-generating compounds (KO_2_ and menadione), and iron depletion (L.-H. Chen, personal communication). Δ*nps*6 failed to produce siderophore, a low-molecular organic compound involved in acquiring iron from the environment [197, 198]. In nearly all living cells, iron required for numerous metabolic functions and electron transfer processes plays a vital role for cell proliferation and survival [199]. When starved for iron, microorganisms secrete siderophores to solubilize and extract iron. All fungal siderophores (rhodotorulic acid, fusarinines, coprogens, and ferrichromes) that have been identified contain hydroxamates and are synthesized from an unconventional amino acid, L-ornithine [197, 200, 201]. NPSs function to synthesize linear or cyclic peptides without the aid of ribosomes, adding D- or L-amino acids, proteins, nonproteins, hydroxyl acids and ornithine into nonribosomal peptides. Many of these peptides have medicinal, pharmaceutical, or agricultural values [202]. AM-toxin produced by the apple pathotype of *A. alternata*, HC-toxin produced by race 1 of the maize pathogen, *Co. carbonum*, and enniatin produced by *Fusarium *spp. are all nonribosomal peptides [7, 203, 204]. 

Many *Alternaria* species produce and excrete dimethyl coprogen siderophores [205–208]. Coprogen contains a diketopiperazine ring (dimerium acid), in which two *N* ^5^-acyl-*N* ^5^-hydroxy-ornithine units are joined by a peptide bond [198]. The third acyl ornithine unit is linked to the ring via an ester bond. NPSs are involved in assembling three *N* ^5^-acyl-*N* ^5^-hydroxy-ornithine units, which are the immediate precursors of hydroxamate siderophores. Fungi often have multiple *NPS* genes; each encodes a polypeptide with discrete domains—AMP-binding adenylation (A), thiolation (T) or peptidyl carrier protein (ACP), and condensation (C) domains that are organized as a module [209–212]. The adenylation domain is required for recognition and activation of amino acid substrate. The thiolation domain is involved in 4′-phosphopantheine binding. The condensation domain is involved in the formation of a peptide bond and elongation and release of the newly synthesized peptide. The number and order of modules in an NPS affect the length and structure of nonribosomal peptide. 

Δ*yap*1 and Δ*nps*6 mutants of *A. alternata* displayed varying levels of hypersensitivity to H_2_O_2_ and superoxide-generating compounds. Δ*nps*6 mutant is less sensitive to ROS than Δ*yap*1, displaying an increased sensitivity to the test oxidants only when applied at higher concentrations: H_2_O_2_ (≥0.2%), KO_2_ (≥20 mM), and menadione (≥5 mM). However, elevated sensitivity to H_2_O_2_ seen in Δ*nps*6 or Δ*yap*1 mutant was alleviated by adding ferric iron into the medium, implicating an important role of iron and siderophore-mediated iron acquisition in the ROS resistance. We have observed that expression of the *AaNPS6* gene was significantly downregulated in fungal mutant lacking *YAP1*. Deletion of *YAP1* in *A. alternata* also reduced the production of siderophores. Moreover, the rescued strain expressing a functional copy of *YAP1* accumulated wild-type level of siderophores and *AaNPS6* gene transcript. Expression of *AaNPS6* and production of siderophores were also down-regulated in fungal strain lacking HOG1 or NOXA (L.-H. Chen and S.L. Yang, personal communication), confirming a close linkage between iron acquisition and ROS resistance. The wild-type strain of *A. alternata*, when grown under iron-depleted conditions, produced barely measurable catalase and SOD activities (L.-H. Chen, personal communication). Both antioxidant activities were detectable in *A. alternata* cultured under iron-rich conditions. Hence, we concluded that the increased sensitivity to oxidative stress and the reduced pathogenicity seen in Δ*yap*1, Δ*hog*1, Δ*noxA*, or Δ*nps*6 were seemingly due to the decreased ability of oxidative stress-detoxifying enzymes. 

Genetic analyses revealed that siderophore produced by NPS6 is required for full virulence of the tangerine pathotype of *A. alternata*. This is likely due to the inability of Δ*nps*6 mutant to detoxify toxic ROS efficiently. Siderophores are also required for fungal pathogenesis in *A. brassicicola*,* As*.* fumigatus*, Co*. heterostrophus*, *Co. miyabeanus*, *F. graminearum*, and *M. grisea* [208, 210, 213, 214]. However, siderophore is not required for pathogenesis of the basidiomycete maize pathogen, *U. maydis* [215]. It will be of great interest to determine if NPS6 is also regulated by YAP1 and HOG1 in other fungal species. 

## 9. Conclusions

**Figure 4:**
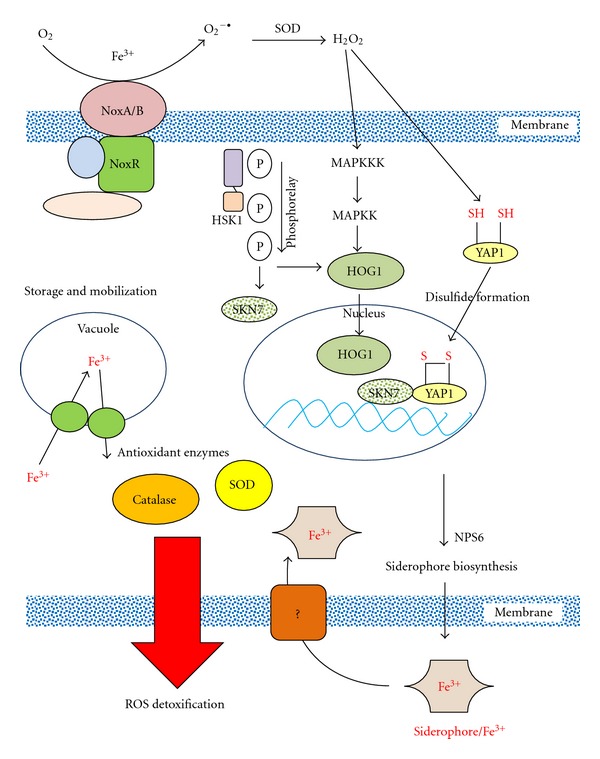
Schematic illustration of signaling pathways leading to ROS detoxification in the tangerine pathotype of *A*. *alternata*. H_2_O_2_ produced by the membrane-bound NADPH oxidase (NOX) complex plays a central role in the activation of genes responsible for ROS resistance. Upon exposure to ROS, YAP1 forms disulfide bonds between two conserved cysteine residues, undergoes conformation changes, and is transported into the nucleus where YAP1 regulates the expression of numerous genes associated with environmental stress. The YAP1 and SKN7 redox-responsive regulators, the HOG1 mitogen-activated protein (MAP) kinase, the NPS6-mediated siderophore biosynthesis, and the NOX complex are required for ROS detoxification. *NPS6* encoding a nonribosomal peptide synthetase is required for the biosynthesis of siderophores, which can extract environmental iron. Iron is stored in vacuoles. Siderophore-mediated iron acquisition plays a critical role if ROS resistance because iron is a major cofactor for the activities of catalase and SOD. HOG1, in cooperation with unknown regulators, is also required for salt resistance. The two-component histidine kinase (HSK1), likely interacting with SKN7, is primarily used for cellular resistance to sugars. Fungicide sensitivity involves HSK1, HOG1, and SKN7.

Based on the observed phenotypes derived from mutants lacking YAP1, HOG1, SKN7, NOX, or NPS6, a regulatory network is assembled to underscore the intricate interplays among these signaling pathways in *A. alternata *(Figure 4). The NOX complex appears to have an important role in the production of ROS, which may act as secondary messages to regulate various metabolic processes in *A. alternata*. The NOX complex is required for transcriptional activation of two important regulators, YAP1 and HOG1, which subsequently regulate the expression of genes encoding the nonribosomal peptide synthetase (NPS6) and perhaps other enzymes involved in the biosynthesis of siderophores as well. SKN7 physically interacts with YAP1, regulating the genes involved in ROS detoxification. Maintaining iron homeostasis is critical for ROS detoxification because of the requirement of iron for antioxidant enzymatic activities. Impairment of the NOX complex, the YAP1 regulator, the HOG1 kinase, or the siderophore-mediated iron acquisition in *A. alternata* impacts its ability to detoxify ROS and to colonize host plant, implicating the importance of ROS detoxification in the successful pathogenesis of *A. alternata*. In addition to detoxifying ROS, *A. alternata* utilizes specialized or synergistically regulated signaling pathways, involved in HSK1, HOG1, and/or SKN7, in response to osmotic stress, fungicides, and other toxic compounds. This cross-interaction between different signaling pathways may have eccentric advantages for integrating cellular responses to a broader spectrum of environmental stimuli.
